# The importance of statistical modelling in clinical research: Comparing multidimensional Rasch-, structural equation and linear regression models for analyzing the depression of relatives of psychiatric patients

**DOI:** 10.1007/s40211-016-0180-3

**Published:** 2016-06-13

**Authors:** Rainer W. Alexandrowicz, Rebecca Jahn, Fabian Friedrich, Anne Unger

**Affiliations:** Department for Applied Psychology and Methods Research, Institute for Psychology, Alps-Adria-University Klagenfurt, Universitätsstr. 65–67, 9020 Klagenfurt, Austria; Clinical Division of Social Psychiatry, Department of Psychiatry and Psychotherapy, Medical University of Vienna, Vienna, Austria

**Keywords:** Rasch Model, Structural Equation Model, Linear Regression Model, Latent regression, Model comparison, Rasch-Modelle, Strukturgleichungsmodelle, Lineare Regressionsmodelle, Latente Regressionsmodelle, Modellvergleich

## Abstract

**Background:**

Various studies have shown that caregiving relatives of schizophrenic patients are at risk of suffering from depression. These studies differ with respect to the applied statistical methods, which could influence the findings. Therefore, the present study analyzes to which extent different methods may cause differing results.

**Methods:**

The present study contrasts by means of one data set the results of three different modelling approaches, Rasch Modelling (RM), Structural Equation Modelling (SEM), and Linear Regression Modelling (LRM).

**Results:**

The results of the three models varied considerably, reflecting the different assumptions of the respective models.

**Conclusions:**

Latent trait models (i. e., RM and SEM) generally provide more convincing results by correcting for measurement error and the RM specifically proves superior for it treats ordered categorical data most adequately.

## Zusammenfassung

**Hintergrund:**

In zahlreichen Studien konnte gezeigt werden, dass die Betreuung schizophrener Patienten durch Angehörige eine große Belastung für die Angehörigen darstellen und zu Depressionen der Angehörigen führen kann. Allerdings unterscheiden sich diese Studien in ihrer statistischen Herangehensweise, was einen Einfluss auf die Ergebnisse haben kann.

**Methodik:**

Die vorliegende Studie vergleicht daher drei verschiedene statistische Modelle, das Rasch-Modell (RM), das Strukturgleichungsmodell (SEM) und das lineare Regressionsmodell an einem Datensatz.

**Ergebnisse:**

Es zeigten sich zum Teil deutliche Unterschiede zwischen den drei untersuchten Modellen, die sich durch deren spezifischen Annahmen erklären lassen.

**Schlussfolgerungen:**

Latent-trait-Modelle (d. h. das RM und das SEM) führen im Allgemeinen zu überzeugenderen Ergebnissen, da sie den Messfehler korrigieren. Das RM erweist sich darüber hinaus als überlegen gegenüber den anderen Modellen, da es ordinalskalierte Daten am angemessensten verarbeiten kann.

## Introduction

Family members frequently care for relatives suffering from schizophrenia. It has been shown that such responsibilities also pose a burden to the caring relatives as well [1, 2]. From Young et al. and Magliano et al. [3, 4] we know that relatives, who care for years for a physically or mentally ill family member, have an increased risk of suffering from depressive conditions. The live-time prevalence of depression increases significantly for relatives of patients suffering from schizophrenia compared to the general population [5]. Similarly, Wittmund et al. [6] found increased depression rates for partners of psychiatric patients in terms of live-time prevalence as well as one-year and four-weeks prevalence.

Although we know that relatives of patients suffering from schizophrenia have an increased risk of depression, little is known regarding possible predictors. On a more general level (i. e. including not only schizophrenia but other psychiatric diagnoses as well), the illness severity has been identified to be a strong predictor for the relatives’ depression [6]. Hobbs [7] reported that mothers, who live in a common household with a schizophrenic patient, have an increased risk of depression when lacking social support. Krautgartner et al. [8] found a correlation of relatives’ depression and the number of previous admissions of the patient and with negative symptomatology. Further, negative symptoms of the patient have been shown to be related to the subjective burden of the relatives [9]. The studies considered so far have taken one relative into account, which is in many cases the mother [10], thus impairing conclusions on a more general level. In contrast, Friedrich et al. [11] collected data of both parents. They found unmet needs of parents to be an important predictor of the caregiving burden. Moreover, the study revealed that symptoms of schizophrenia also predict the parents’ depression [12]. Considering all these studies, we dispose of ample evidence that caregiving relatives of a patient suffering from schizophrenia are at risk of becoming depressive.

## Research question

The present study focusses on the important methodological aspect that various statistical techniques have been applied to investigate the research questions mentioned above, differing strongly in their assumptions and which information contained in the data they use: In [1, 4–9, 11], linear regression models were applied, studies [3, 6, 8] used logistic regression (which, in short, is a generalization of linear regression modelling if the dependent variable is dichotomous; studies [2] and [10] did not perform statistical analyses). In contrast, Alexandrowicz et al. [12] applied a latent variable approach using a multidimensional Rasch Model. The present study will, therefore, analyze, whether the chosen statistical technique differs with respect to the outcome of a study.

For that purpose, three fundamental methodological approaches are taken into account: A multidimensional Rasch model has been applied for identifying the effects reported in Alexandrowicz et al. [12]. For a comprehensible introduction to the principles of Rasch models see, for example, de Ayala [13], a comprehensive technical treatment provide Fischer and Molenaar [14], and for the multidimensional case see von Davier and Carstensen [15]. Basically, Rasch models seek to describe individuals and items with respect to a common underlying dimension (in our case depressiveness). Multidimensional Rasch models and their extension to latent regression modelling are a comparatively young technique. Prior to their availability, Structural Equation Modelling (SEM [16, 17]) and Linear Regression Models (LRM [18]) were the prevailing techniques used in studies like the ones described above. It is therefore interesting to analyze, whether these three different methodological approaches yield the same, similar, or maybe entirely different results. This point is crucial, as different methods should not induce contradicting results, otherwise we would have to deal with method artifacts. Therefore the present study investigates the same research question, i. e. how the patients symptoms of schizophrenia can predict mothers’ and fathers’ depression, using the three different statistical methods, Rasch-model, SEM and LRM.

## Methods

In order to preserve comparability of the three modelling approaches, we use the data set from [11, 12]. This study comprised 101 patients suffering from schizophrenia along with both their mothers and fathers. Parents completed the “Beck Depression Inventory” (BDI [19]). Patients were assessed by means of the “Positive and Negative Syndrome Scale” (PANSS [20]), which is composed of three subscales covering positive symptoms (PANSS-P, e. g., hallucinatory behavior, excitement, or grandiosity), negative symptoms (PANSS-N, e. g., emotional withdrawal or difficulties in abstract thinking), and general symptoms (PANSS-G, e. g., anxiety, disorientation, or lack of judgement and insight). All scales were corrected for the patient’s age, gender, number of previous hospital admissions, and duration of illness.

The major concern of Alexandrowicz et al. [12] was the latent structure, i. e., how the three symptom scales covered by PANSS predict mothers’ and fathers’ latent depression scores (BDI). Additionally, a background model corrected for influences as regards the patients’ gender, age, number of admissions, and duration of illness. The results of the Rasch-based analysis are taken from the original paper (for details see [12]).

### The Rasch Approach

Three aspects of Rasch models are crucial to our present effectuations. First of all, there is the notion of a latent scale. The responses we obtain in the questionnaire are considered as manifestations of a latent (i. e., not directly observable) propensity to respond. For example, the BDI used in [12] sets out to measure depression. The Rasch Model provides us with (a) one parameter estimate per individual per scale (i. e. depressiveness) describing his or her location on this latent dimension and (b) one parameter for each category threshold separating adjacent response categories of each item on that scale. These thresholds mark the locations on the latent scale, as of which individuals are more likely to choose the higher of two adjacent categories. For example, if an individual shows a person parameter of 1.3 and the threshold between category 2 and 3 of an item is 0.8, then the person is expected to choose category 3; if, in contrast, the person showed a parameter of, say, 0.3, then he or she is more likely to choose category 2.

By presenting several items per scale, we obtain several manifestations of an individual’s location on that latent scale. Parameter estimation can best be understood as a kind of triangulation method seeking that location of an individual on the latent scale, which makes all observed responses most likely to have appeared (hence, the most important parameter estimation method is termed maximum likelihood method). Such a modelling approach has the advantage of minimizing the effect of random fluctuations (termed random errors), inevitably occurring when we apply questionnaires like the BDI or the PANSS in clinical assessment (cf. [21]).

The second aspect relates to the response format. Most of the scales applied in clinical research use a Likert-type response format, i. e., respondents are presented a question or a statement and choose the most appropriate response category from a list of response alternatives. It is typical for clinical scales that these alternatives are ordered, for example, the PANSS scales use 0 = absent, 1 = minimal, 2 = mild, 3 = moderate, 4 = moderate severe, 5 = severe, and 6 = extreme. Although categories are numerically coded, the responses are ordinal, i. e., we do not assume that the category codings (0, 1, 2 …) are equidistantly spaced in the latent dimension: Consider four respondents, A to D, choosing categories A = 0 (absent), B = 1 (minimal), C = 5 (severe), and D = 6 (extreme). While A/B and C/D differ by exactly one unit on the manifest scale, they do not necessarily differ by the same amount in their symptom severity (as an interval scale would imply). Rather, these responses only allow for establishing a rank order (B expresses severer symptoms than A, etc.; note that the coding of negatively worded items has to be reversed so that higher scores imply a higher location of the respondent on the latent scale). However, statistical procedures, such as the mean, the (co-)variance, the Pearson correlation coefficient, or a linear regression model do in fact assume the responses themselves to be interval scaled and may therefore result in misleading conclusions. In contrast, Rasch models treat such data indeed as ordered categorical and do not evaluate the sum of the codings, but rather use the category frequencies. Furthermore, no distributional assumptions regarding the item category frequencies are required in contrast to other common statistical methods requiring normally distributed data (e. g., that the category frequencies resemble a normal distribution with code 3, on a scale from 0 to 6, appearing most often and decreasing frequencies the more we approach the extremes).

And third, in a multidimensional case like the one considered here, five dimensions are considered (PANSS positive, PANSS negative, PANSS general, depression of the mother, and depression of the father). For each of these 5 dimensions, a latent scale of its own is established and we obtain 5 separate estimates of an individual’s location (person parameter) on each of these latent scales. Because the five dimensions are estimated simultaneously, we are able to draw conclusions concerning their interplay. This regards both correlations between the constructs and regression structures, i. e., selected latent constructs serve as predictors (also termed independent variables, in our case the three PANSS-scales), while other latent constructs form the criteria (or dependent variables, in our case mothers’ and fathers’ depression).

The multidimensional Rasch Model was used in the formulation of the Multidimensional Random Coefficients Multinomial Logit Model (MRCMLM [22, 23]). This model allows for estimating the parameters of a Rasch Model according to the principles outlined above and further extends the RM by additionally considering a so-called background model. A background model uses information on the respondents which is not covered by the items analyzed (in our case the patient’s age, gender, number of previous admissions, and duration of illness) and eliminates the influence of these variables. The resulting parameter estimates are therefore corrected for the effects of such background variables. Note that Rasch models in general allow for estimating the parameters without making distributional assumptions. However, the MRCMLM uses an algorithm, which assumes the person parameters to be normally distributed.

**Fig. 1:**
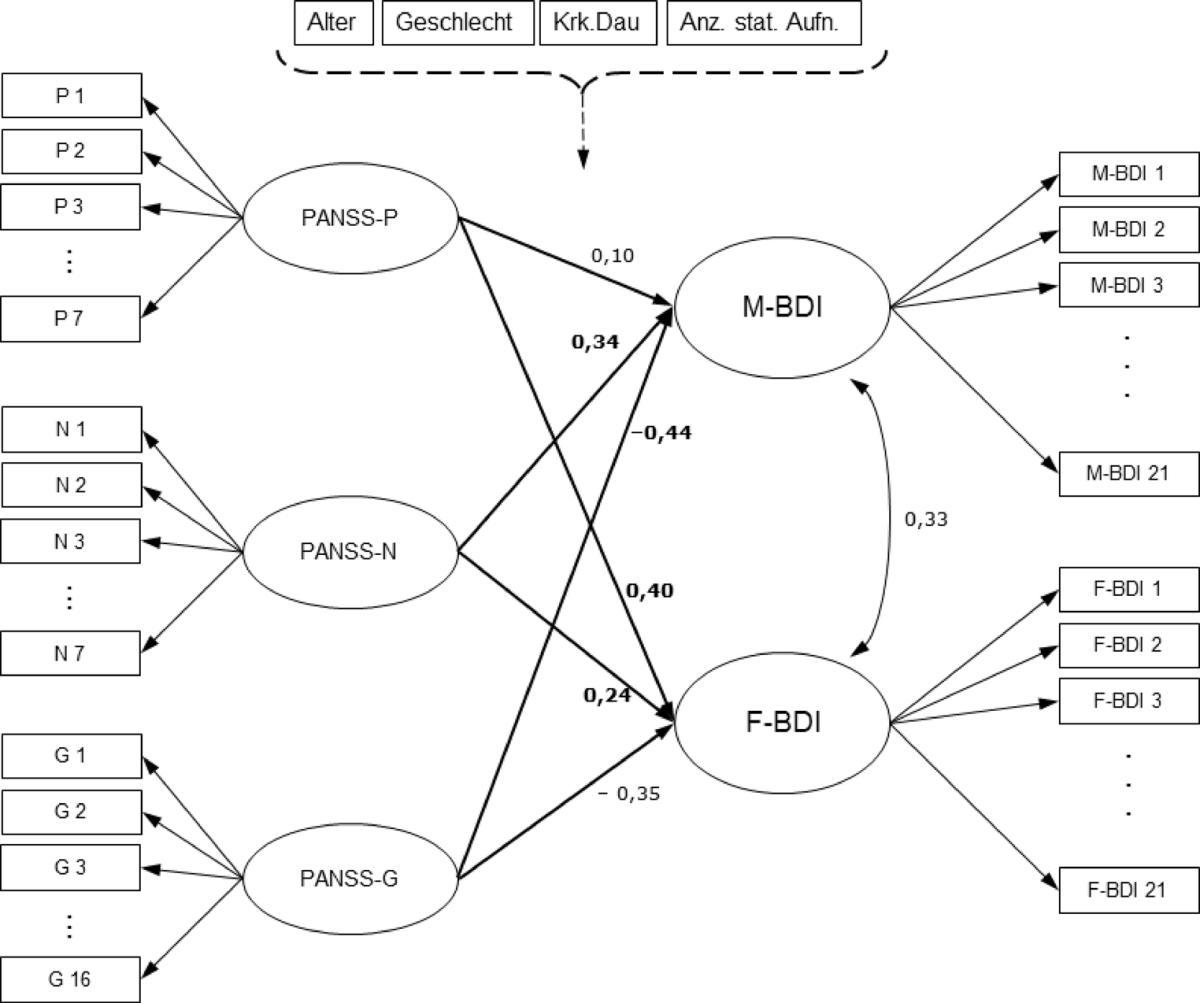
Outline of the MRCMLM- and the SEM-approach

Fig. 1 (which is adapted from Alexandrowicz et al. [12]) shows the outline of the model.

### The SEM Approach

A Structural Equation Model allows for modelling the correlations and regressions of latent variables based on sets of manifest items. Thus, SEMs share some similarity with the multidimensional Rasch models described above. However, in their original and most frequently applied form, SEMs require the data to lie on an interval scale and to follow a multivariate normal distribution. Extensions to ordinal data and corrections for non-normal data have been introduced, but these are rarely applied. One may envisage SEMs as a kind of extended factor analysis (cf. [23, 24]), in which we restrict both the loadings of the items on the latent factors and the interplay of the latent factors based on substantive reasoning. The present analysis contrasts the Rasch-based approach of Alexandrowicz et al. [12] to such a “traditional” SEM, i. e., using response category codings as if they were interval scaled.

A corresponding structure to the model of Alexandrowicz et al. [12] has been established in the SEM framework, i. e., the items of the scales were again covered by one latent factor each and the regression paths according to the research question (i. e., BDI-M/F on PANSS-P/N/G) were estimated in an analogous fashion. The SEM does not explicitly model a background model like the MRCMLM. However, it was realized by regressing the five latent factors (PANSS-P/N/G and BDI-M/F) on the four background variables (age, gender, number of previous admissions, and duration of illness). Thus, the structure depicted in Fig. 1 applies to the SEM approach as well.

One major difference between the SEM and the MRCMLM can be seen in the fact that the SEM relies on the covariances (i. e., unstandardized correlation coefficients) between all manifest variables (e. g., the items of a scale), while the MRCMLM uses the observed frequencies of each response category (hence the former treats the responses as interval scaled and the latter as ordinal). Moreover, a SEM assumes the manifest responses of all items to follow a multivariate normal distribution, while the MRCMLM makes this assumption only for the latent distribution of the person parameters. Note that modified techniques have been developed to apply a SEM to ordinal responses as well by using correlation measures for ordinal data. But these modifications still assume all pairs of items to follow a (then latent) bivariate normal distribution, and, moreover, they involve an extra estimation step, which can result in miscalculations.

### The LRM Approach

In contrast to the two latent variable approaches introduced so far, one could also consider choosing the more straightforward and simpler Linear Regression Model. This approach does not attempt to estimate a location on a latent dimension but rather relies on the sum of the category codings of the items forming a scale, thus disregarding the limitations of ordinal codings. Again, reversed items have to be recoded and for some scales, the manual prescribes specific transformations to gain a weighted score. Furthermore, taking the sum across items implicitly assumes all items to measure one and the same trait, i. e., unidimensionality is tacitly presumed. Moreover, normal distribution of the residuals and homoscedasticity are required (cf. [18]).

**Fig. 2:**
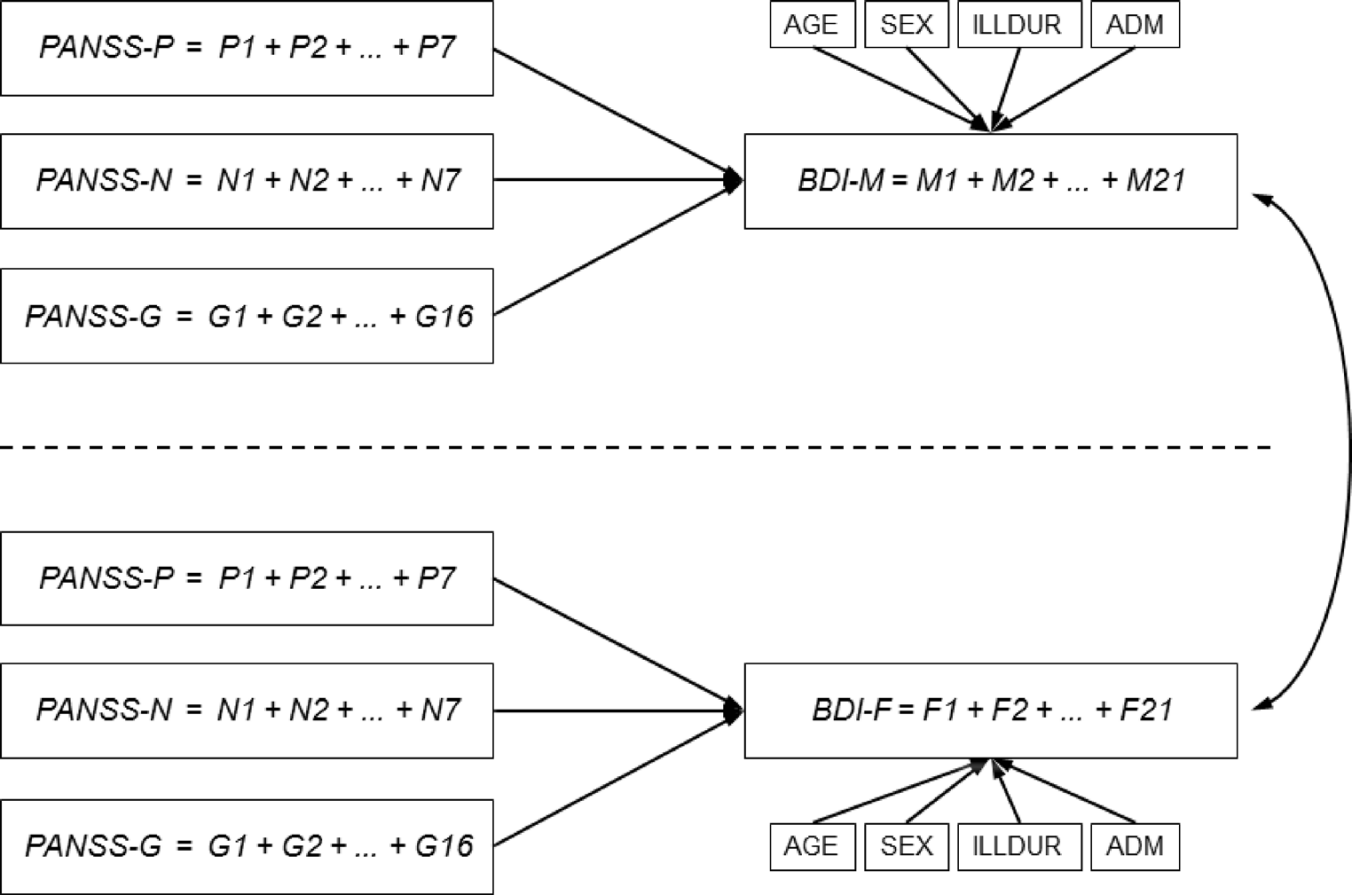
Outline of the LRM-approach

To answer the research question by applying an LRM, we first calculated the five sums of the items of each scale, yielding a score for PANSS-P, PANSS-N, PANSS-G, BDI-M, and BDI-F. None of the scales involved uses reversed items, therefore no recoding was necessary. LRM differs fundamentally from the latent variables approaches realized by the MRCMLM and the SEM, which both constitute *multivariate* (latent) regression models. This means that we consider more than one *dependent* variable at a time (nevertheless, all three models are *multiple* models, i. e., include more than one *independent* variable). Therefore, with LRM, we cannot estimate all parameters of interest in one run. Rather, we have to formulate two separate models, one for the dependent variable BDI-M and one for BDI-F. Linear regression models also allow for considering background variables, yet in a different way by entering them as additional predictors. This model will be labelled LRM1 and Fig. 2 outlines it.

However, LRM1 does not fully comply with the latent modelling approaches (i. e., the MRCMLM and SEM), because the background variables are only related to the respective dependent variable (i. e., BDI-M and BDI-F), but not to the three predictor variables (PANSS-P/N/G). Furthermore, the correlation of the two BDI-variables would not be corrected for any of the predictors or background variables because of the separate modelling approach.

We may emulate the background models in the context of a simple regression model in a way, which is more similar to the MRCMLM or SEM technique, but that requires some quite uncommon steps: We first build the background model, i. e., regress the five constructs of interest (PANSS-P/N/G and BDI-M/F) on the four background variables (age, gender, number of previous admissions, and duration of illness) and store the *residuals* of each of these five models. These residuals contain all information of the five constructs of interest not explained by the background variables. In a second step, we estimate the two regression models we are actually interested in, i. e., BDI-M and BDI-F on PANS-P/N/G, by making use of these residuals. Furthermore, we can calculate the correlation of the two depression scores in the same fashion (which, from a more technical point of view, is a simple partial correlation coefficient). This model will be labelled LRM2.

### Technical details

The present study uses the data set of Alexandrowicz et al. [12], details regarding the scales and the sample can be found there. Note that the items had been dichotomized for the Rasch analysis (BDI: 0/1–3; PANSS: 1–2/3–6) for technical reasons, because a total of 72 items with 4 (BDI) and 6 (PANSS) response categories would require a larger sample to warrant stable parameter estimates. The results reported by Alexandrowicz et al. [12] are contrasted to the SEM and the regression analysis. The SEM and the LRM analyses have been performed with R [25], for a comprehensible introduction see [26]. For the SEM analysis we used the R‑package lavaan [27]. The significance level was set to 0.05 for all analyses.

## Results

The results will be presented according to the three major sections of analysis, i. e., (a) the regression coefficients of the background model, (b) the regression paths of the PANSS-scales upon the two BDI-scales, and (c) measures of model adequacy. Within each section the three modelling approaches will be contrasted.

### The Background Model

Tab. 1 presents the regression coefficients of the background variables upon the five scales. Each cell shows the respective coefficient for the MRCMLM, the SEM and the two regression models.

**Table 1:** Background model: Regression coefficients and standard errors

		P	S.E.	N	S.E.	G	S.E.	M	S.E.	F	S.E.
INT	MRCMLM	−1.22	*0.91*	−2.85	*0.81*	−2.25	*0.76*	−0.50	*0.92*	**−2.85**	*0.75*
	SEM	[1]	*–*	[1]	*–*	[1]	*–*	[1]	*–*	[1]	*–*
	REG1	[2]	*–*	[2]	*–*	[2]	*–*	[2]	*–*	[2]	*–*
	REG2	**11.35**	*2.63*	**14.56**	*3.60*	**25.10**	*5.12*	**9.08**	*3.52*	2.48	*2.97*
AGE	MRCMLM	0.00	*0.04*	0.04	*0.03*	0.03	*0.03*	0.05	*0.04*	**0.06**	*0.03*
	SEM	0.07	*0.29*	0.20	*0.27*	0.14	*0.13*	−0.10	*0.12*	0.12	*0.08*
	REG1	[3]	*–*	[3]	*–*	[3]	*–*	−0.01	*0.14*	0.03	*0.11*
	REG2	0.10	*0.10*	0.19	*0.14*	0.26	*0.20*	0.02	*0.13*	0.07	*0.11*
GEN	MRCMLM	−0.15	*0.45*	0.57	*0.40*	0.20	*0.38*	0.83	*0.46*	0.47	*0.37*
	SEM	0.02	*0.02*	0.04	*0.02*	0.01	*0.01*	0.00	*0.01*	0.00	*0.01*
	REG1	[3]	*–*	[3]	*–*	[3]	*–*	−0.63	*1.61*	1.77	*1.29*
	REG2	−0.49	*1.18*	0.83	*1.61*	0.93	*2.29*	−0.47	*1.59*	1.86	*1.30*
ILL	MRCMLM	−0.01	*0.05*	−0.03	*0.05*	−0.04	*0.05*	**−0.17**	*0.05*	**−0.11**	*0.04*
	SEM	0.00	*0.03*	**−0.09**	*0.03*	−0.03	*0.02*	0.01	*0.02*	0.00	*0.01*
	REG1	[3]	*–*	[3]	*–*	[3]	*–*	−0.04	*0.20*	0.10	*0.15*
	REG2	−0.09	*0.14*	**−0.50**	*0.19*	**−0.66**	*0.27*	−0.12	*0.19*	0.03	*0.15*
ADM	MRCMLM	0.03	*0.08*	−0.04	*0.07*	0.12	*0.07*	0.13	*0.08*	**0.19**	*0.06*
	SEM	0.03	*0.05*	0.08	*0.05*	**0.08**	*0.03*	0.01	*0.02*	−0.01	*0.02*
	REG1	[3]	*–*	[3]	*–*	[3]	*–*	0.19	*0.31*	−0.18	*0.24*
	REG2	**0.40**	*0.20*	**0.57**	*0.27*	**1.73**	*0.39*	0.23	*0.28*	−0.11	*0.22*

Rows = predictor variables, columns = dependent variables

Bold entries indicate coefficients significantly different from zero

[1] In the SEM context, all latent variables are centered, therefore no intercept is estimated

[2] In the REG2-model, no separate intercept is estimated for the background variables

[3] In the REG1-model only BDI-M and BDI-F serve as dependent variables, hence the PANSS-scales are not regressed on the background variables

*P* PANSS-P, *N* PANSS-N, *G* PANSS-G, *M* BDI-M, *F* BDI-F, *S.E.* standard error, *INT* intercept, *AGE* age of patient, *GEN* gender of patient, *ILL* illnes duration of patient, *ADM* number of admissions of patient

The background variables differ in their influence upon the central measures (PANS-P/N/G and BDI-M/F). With the Rasch Model (MRCMLM), we find four effects: a slight (but significant) influence of the patient’s age on the fathers’ depression, of the duration of illness on the depression of both mothers and fathers (both show inverse relations, i. e., the longer the patient suffers from schizophrenia, the weaker are the parents’ depression symptoms), and of the number of previous admissions, again on the fathers’ severity of depression. When using the SEM, we find two effects, first duration of illness weakly predicting the negative symptoms (inversely, i. e., with persisting schizophrenia the negative symptom score decreases slightly), and second the number of previous admissions predicting the general symptomatology (both to a very weak extent). When we consider the linear regression approach (LRM1), only the effects of the background variables upon the two depression measures can be evaluated, none of which showed a significant influence. Considering the enhanced background model approach (LRM2), mimicking the full background model of a SEM or an MRCMLM, we find the number of previous admissions significantly predicting all three PANS-scales. The intercept of all models reflects the grand mean and is therefore not of substantial interest.

### The (Latent) Regression Model

After correcting for the background variables, we turn to the regression model (which conforms to the structural model in terms of SEM), constituting the central part of the analysis. Again we identify considerably differing results (cf. Tab. 2).

**Table 2:** Coefficients of the (latent) regression model and correlation of the two depression measures

		BDI-M	S.E.	BDI-F	S.E.
INTERCEPT	MRCMLM	−1.59	*0.29*	−1.74	*0.26*
	SEM	[1]	*–*	[1]	*–*
	REG1	7.47	*4.02*	−0.77	*3.31*
	REG2	0.04	*0.71*	−0.004	*0.57*
PANSS-P	MRCMLM	0.11	*0.19*	**0.4**	*0.17*
	SEM	−0.01	*0.05*	0.01	*0.03*
	REG1	−0.03	*0.18*	0.19	*0.15*
	REG2	−0.03	*0.18*	0.19	*0.14*
PANSS-N	MRCMLM	**0.35**	*0.13*	**0.24**	*0.12*
	SEM	**0.12**	*0.05*	0.04	*0.03*
	REG1	0.19	*0.14*	0.19	*0.11*
	REG2	0.19	*0.14*	0.19	*0.11*
PANSS-G	MRCMLM	**−0.44**	*0.21*	−0.35	*0.19*
	SEM	−0.07	*0.11*	0.01	*0.07*
	REG1	−0.03	*0.12*	−0.06	*0.1*
	REG2	−0.03	*0.12*	−0.06	*0.09*

[1] Because in the SEM context all latent variables are centered no intercept is estimated

*S.E.* standard error

The MRCMLM shows that the positive and the negative symptoms significantly predict the fathers’ depression and that the negative and the general symptoms significantly predict the mothers’ depression. The last coefficient (PANSS-G/BDI-M) displays a negative sign (i. e., the more general symptoms a patient reports the less depression his or her mother reports), which seems unexpected at first sight. But it can be explained by the fact that the influence of the duration of illness has already been partialled out in the background model and the remaining variability can be explained by familiarization with the symptomatology (see [12] for more details regarding the clinical implications of these results). Looking at the SEM result, we can only identify the significant path from negative symptoms to the mothers’ depression (which conforms to the MRCMLM result). The regression based approach has not revealed any significant coefficients (LRM1), not even after partialling out the background variables (LRM2).

The correlation of the (latent) dependent variables (BDI-M and BDI-F) is similar across all four models under consideration: MRCMLM: 0.352, SEM: 0.442, LRM1: 0.372, and LRM2: 0.384, all being significant.

### Model Fit

All models provide for an inspection of model adequacy, some of which are outlined here. From the MRCMLM we obtain indices of item fit, expressing the adequacy of the mathematical function (which is the logit function, see [13, 14]) used to link the category frequencies to the latent scale (accompanied by a convenient graphical representation). Furthermore, the scale reliability tells us, whether the items allow for a sufficient differentiation of respondents regarding their location on the latent scale (this index is roughly comparable to the classical reliability index, yet founded on a more sophisticated mathematical model). Both measures indicate unsatisfactory model fit as reported by Alexandrowicz et al. [12]. From the item based indices one could derive indications for which items are prone to misfit and should therefore undergo more detailed psychometric exploration. Furthermore, the program outputs an *R*^2^-statistic of the latent regression models, indicating the amount of variance of the latent dependent variables (BDI-M and BDI-F), which is explained by the latent predictors (PANSS-P/N/G). These are 0.08 for BDI-M and 0.11 for BDI-F. Although these values seem very low from a general point of view, we have to keep in mind that the model has not been set up to explain the depression in its entirety, but to display the role of the symptoms only.

The SEM is known for providing a plethora of item fit measures (cf. [28]). We want to inspect some of the most commonly used ones: *χ*^2^ = 5,524; df = 2,745; p < 0.05; *χ*^2^/df =2.013; CFI = 0.361; TLI = 0.338; RMSEA = 0.106. All measures indicate poor model fit, yet the chi-square-to-df-ratio of approximately 2 seems within an acceptable range according to Bollen [29, p. 278]. If we would want to further explore the reasons for the misfit, we could, for example, analyze the factor loadings and the residual covariance matrix to obtain further hints on which items prove troublesome – an endeavour, which is outside the scope of the present study. The *R*^2^-values are 0.09 for BDI-M and 0.06 for BDI-F.

When we turn to the linear regression model(s), we also encounter a large number of techniques to assess model fit (cf. [30–32]). The *R*^2^-statistics in model LRM1 are 0.04 for BDI-M and 0.10 for BDI-F, and in LRM2 the respective values are 0.03 and 0.07. Hence, the explained variance is of similar size compared to the other two approaches.

Routines to assess the fit of regression model focus on the score entered into the model rather than on how items establish such a score. Alternatively, one might further apply an item-oriented measure like the (corrected) item-total-correlation. This is not part of the regression model as such, but constitutes an auxiliary analysis step (hence not requiring to distinguish LRM1 from LRM2). It allows for a comparison of our three modelling approaches.

**Fig. 3:**
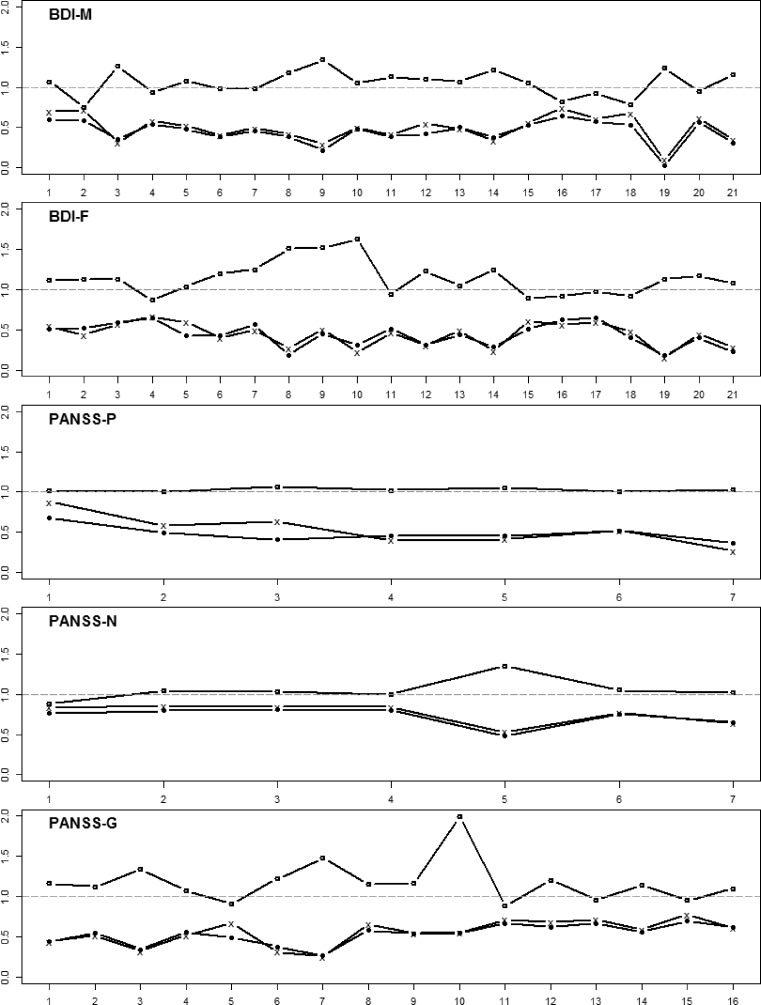
Item-based fit-measures for the five scales; *circles*: outfit indices of the MRCMLM; “*x*”: factor loadings of the SEM; *bullets*: corrected item-total-correlation

Fig. 3 shows the corrected item-total-correlation (*r*_it_; indicated with bullets), the factor loadings of the SEM (*λ*_i_; indicated with “x”), and the outfit index (indicated with circles) of the MRCMLM for all five (latent) scales. Note that the first two (*r*_it_ and *λ*_i_) share the same range of zero to one, with values close to one indicating item fit. In contrast, the optimal value of the outfit index is one and deviations from one in both directions indicate misfit.

The plots show an interesting pattern: The measures *r*_it_ and *λ*_i_ agree remarkably, and the outfit values show a fairly similar pattern, just mirrored. Hence, one would, by and large, identify mostly the same items as problematic, independently of the applied technique. Especially for the regression and the SEM-based analysis, we obtain virtually the same information. Slight differences have emerged in the PANSS-P scale, where the outfit measure indicates excellent fit for all items, while the other two show somewhat mediocre fit (except for the first item). Nevertheless, all three techniques appear to highlight similar issues.

## Discussion

In this study, three different statistical modelling approaches have been contrasted – the comparably new latent regression routine in the MRDMLM-framework, the somewhat older SEM-approach and a “classical” Linear Regression Model (in two variants).

The results of the three analyses differ considerably. Regarding the background model, we have identified a number of differences: While the straightforward regression approach (LRM1) has not identified an influence of the background variables (patient’s age, gender, number of previous hospital admissions, duration of illness) upon the dependent variables BDI-M and BDI-V, the more sophisticated residual-based approach (LRM2), which also partials out the influence of the background variables upon the predictor variables (PANSS-P/N/G), has revealed five significant influences (duration of illness upon positive and negative symptoms, and number of previous admissions upon all three symptom scales). This is a first indication that the more complex model captures more of the information contained in the data. Nevertheless, both approaches have to be considered as basic, because they do not take the measurement error into account. The SEM, which takes measurement error into account by forming latent scales, shows two significant effects of the background variables, first of the duration of illness upon PANSS-N and second of the number of previous admissions upon the PANSS-G. The MRCMLM establishes three significant effects of the background variables: duration of illness significantly predicts the two dependent variables BDI-M and BDI-F and the number of admissions again on BDI-F. Researchers applying a plain regression model usually prefer a formulation like the one LRM1, i. e., they use background variables in the role of additional predictors. This approach however has not revealed any effect of the background variables. The more complex approach of LRM2, i. e., estimating an auxiliary model and continuing with the residuals, is rarely chosen, the authors are at least not aware of such an application. We may therefore state as a first result that the LRM approach is prone to overseeing effects. The two models using latent constructs and therefore correct for measurement errors are in contrast able to detect influences of the background variables and can thus exclude these variables from the regression of interest (i. e., of BDI-M/F on PANSS-P/N/G). However, results of SEM and MRCMLM differ, an issue we will elaborate on further below.

Switching to the regression of BDI-M/F on PANSS-P/N/G, which is the major focus of the analysis, we find no significant effect at all with the plain regression model (i. e., LRM1 and LRM2), one significant regression coefficient with the SEM (BDI-M on PANSS-N), but four significant effects with the MRCMLM (PANSS-P upon BDI-F; PANSS-N upon both BDI-M and BDI-F; PANSS-G upon BDI-M). These differences strongly indicate that taking the measurement error into account allows for better detecting effects of the patients’ symptomatology upon the parents’ depression. The different results of SEM and MRCMLM could partially be due to the fact that the items had to be dichotomized in order to successfully apply the MRCMLM, while the original codings were used in the SEM. But interestingly, although dichotomization causes a loss of information, the MRCMLM results are more differentiated than those of the SEM. This raises the question, whether the number of response categories of the instruments considered here is in fact optimal, as over-differentiation may also cause statistical noise. Another reason for the differences between SEM and MRCMLM could be that we applied the SEM in a mathematically flawed (yet common) way by using the ratings as if they were interval scaled although they are only ordinal. But because this is still the way SEMs are usually applied (e. g., [33] for the BDI or [34] for the PANSS), we deliberately decided to keep up with this tradition. Moreover, if one decided to apply the SEM more appropriately by treating the data as ordinal, the assumption of normally distributed item categories still applies, which is not the case when using a Rasch Model in general (and to a limited extent in case of the MRCMLM). In contrast, the necessity to dichotomize diminishes automatically with increasing sample size. We therefore prefer the results of the MRCMLM.

In addition to interpreting the various model coefficients, we also have to consider the model fit, i. e., whether the applied models are capable of adequately describing the data. The fit measures of all three models indicate that further refinements seem appropriate. First of all, one takes a look at the R^2^ values of the LRMs, which reveal the portion of variance of the dependent variable that can be explained by the independent variables. The R^2^ values could be considered low at first sight, but remember that the original study did not set out to fully explain parents’ depression, which would require a much more complex study design. Rather, the intention was to specifically find effects of the patients’ symptomatology upon the parents’ depression, which is a much more focused research question. Therefore, results are in fact interesting from a clinical point of view.

One highly relevant question of significance testing is power, i. e. how large a sample is required to detect an effect of substantive interest with given limits of an error of the first and of the second kind. However, while power analysis is fairly easy for univariate analyses (e. g. the *t*-test or ANOVA models), the models considered here exhibit much more complexity. We estimate a large number of coefficients, each of which may be tested for differing significantly from zero. Moreover, also the model fit can be assessed by means of a significance test. Because of this complexity, power analysis is not that straightforward as, for example, in an ANOVA. Solutions exist for each of the models considered here (cf. [35–37] for the RM; [38] for the SEM, or [18] for the LRM). But due to the complexity of the models, a direct comparison of power (e. g., in the sense of [39]) is difficult to achieve, as it depends on various details of the actual model formulation.

Summarizing the apparent superiority of the Rasch-based approach, we have identified two major factors: First of all, this model involves latent constructs, thus removing measurement error associated with manifest codings. Hence, we use error-adjusted estimates of both the predictor variables (schizophrenia symptoms as covered by the PANSS) and the criteria (depression symptoms as covered by the BDI). Second, the Rasch Model applied in the analysis (the MRCMLM) treats categorical data adequately and does not assume them to lie on an interval scale. Category locations are explicitly estimated and thus reflect their empirical distances as perceived by the respondents. This allows for a much more precise processing of the actual information in the sample. However, when there are many polytomous items to analyze, samples should be somewhat larger compared to applying an LRM.

If the data set is composed of several groups representing different populations, the question arises, whether an instrument allows for equivalent measurements in these groups. Both latent variable models allow for elegantly checking this assumption. In the SEM context, we refer to measurement invariance [40], for an interesting application in the clinical context see [41]. In the RM context, we refer to Differential Item Functioning (DIF [42]).

The LRM approach has shown some clear deficiencies: First of all, it would be remarkably cumbersome to build an adequately complex model (like we did in LRM2), requiring us to first determine the residuals (i. e., control for the background variables) before we could estimate the regression structure of interest. Second, the complex output regarding item- and model fit also requires an extra step by calculating (for example) the item-total-correlation. Hence, such an approach would rarely be applied (the authors are not aware of any published study following such a procedure). Rather, researchers employing LRM would just carry out the more straightforward approach by simply entering the background variables as additional predictors (as we did in LRM1). Moreover, none of the two linear regression models have shown results comparable to those obtained with latent variable models.

## Conclusion

The present study has impressively shown how different modelling approaches may lead to considerably different results. Although the present study considers only one data set, the differences of the results could plausibly be traced back to fundamental differences in the way the three models considered here extract information from the data. Hence, our results convincingly demonstrate, how severely the researcher’s choice of model affects the results of an analysis and that method artifacts cannot be ruled out when using inappropriate statistical tools. The classical approach (LRM) has proven most deficient in our case. The most sophisticated results have been established with a multidimensional Rasch Model (the MRCMLM), which (a) treats ordered categorical data in a most natural way, not making doubtable assumptions regarding the scale, (b) assumes a latent structure to exist behind the manifest variables and thus controls for measurement error, and (c) allows for a multivariate treatment of a complex structure. This Rasch Modelling approach is therefore to be recommended as the gold standard for clinical studies using rating scales.
